# A four-dimensional spatial atlas of spinal cord injury reveals predominant Spp1-integrin signaling driving microglia-fibroblast crosstalk in fibrotic niches

**DOI:** 10.3389/fimmu.2026.1926947

**Published:** 2026-09-11

**Authors:** Liwen Chen, Siqiao Wang, Zhihui Xiao, Shitong Zhao, Wenyong Fan

**Affiliations:** Division of Spine, Department of Orthopedics, Tongji Hospital Affiliated to Tongji University, School of Medicine, Tongji University, Shanghai, China

**Keywords:** fibrotic scar, microglia-fibroblast crosstalk, single-cell RNA sequencing, spatial transcriptomics, spinal cord injury

## Abstract

**Background:**

Spinal cord injury (SCI) leads to fibrotic scarring that blocks axonal regeneration, yet the molecular mechanisms controlling scar formation remain incompletely understood. Microglia and fibroblasts accumulate at lesion sites, but their intercellular communication, spatial organization and therapeutic tractability have not been systematically mapped.

**Methods:**

We integrated spatial transcriptomics, single-cell transcriptomics, bulk RNA sequencing, and microarray profiling across multiple post-injury time points in mouse and rat SCI models. A four-dimensional spatial atlas spanning three spatial dimensions and time was constructed to map microglia-fibroblast interactions. Fibroblast differentiation trajectories were used to establish molecular subtypes and a risk prediction model, validated in independent cohorts and human peripheral blood. NT3-chitosan was tested in a complete transection model with functional and molecular assessments.

**Results:**

The four-dimensional spatial atlas identified Spp1-Itgav/Itgb1 as the dominant microglia-to-fibroblast signaling axis during peak scar consolidation, spatially colocalized with NF-κB and TGF-β transcriptional programs. Fibroblast differentiation-based classification stratified SCI into three subtypes associated with injury severity and immune remodeling. A risk model based on fibroblast differentiation-associated signatures in peripheral blood predicted ASIA grade A with AUCs of 0.883, 0.939, and 0.807 in the total, training, and test sets, respectively. NT3-chitosan treatment was associated with reduced Spp1-Itgav/Itgb1 signaling, diminished fibrotic scarring, and sustained locomotor recovery over 52 weeks.

**Conclusion:**

Microglia-fibroblast crosstalk via Spp1-Itgav/Itgb1 is enriched in regions of fibrotic scar maturation, alongside NF-κB and TGF-β transcriptional activation. The fibroblast differentiation-based framework enables risk stratification and guides scar-targeted interventions, providing a clinically actionable pathway for SCI therapy.

## 1 Introduction

Spinal cord injury (SCI) affects millions worldwide and remains one of the most disabling neurological conditions (1). Decades of clinical trials have failed to produce a therapy that repairs the damaged spinal cord. The scar at the injury site is the principal barrier to regeneration. This scar has two interrelated components: a glial scar of reactive astrocytes and a fibrotic core of activated fibroblasts with dense extracellular matrix. The glial scar has drawn extensive attention, but the fibrotic component remains comparatively overlooked, even though it forms the physical barrier that severed axons must cross to reconnect across the lesion (2, 3). Importantly, moderate reduction of fibrotic scarring, while still permitting wound closure, promotes axon regeneration and functional recovery, making the fibrotic scar a compelling therapeutic target (4, 5).

Recent lineage tracing studies have clarified fibroblast origins in the injured spinal cord. Scar-forming fibroblasts derive from two perivascular cell populations, pericytes and perivascular fibroblasts, distributed heterogeneously across the cord and contributing to scar formation in a region-dependent manner (5). What remains poorly understood is the identity of extracellular signals that instruct these fibroblasts when to activate, where to migrate, and how extensively to deposit matrix. The most plausible source lies in the surrounding immune microenvironment. Microglia, the resident macrophages in central nervous system (CNS), activate massively after injury and accumulate precisely where fibroblasts congregate (6). Emerging evidence shows that microglia release sphingosine-1-phosphate to promote pericyte migration and fibroblast transition, indicating that these two cell types do not act in isolation (6). Yet the broader landscape of microglia–fibroblast communication, its temporal evolution, spatial organization within the three-dimensional lesion architecture, and therapeutic tractability remain largely uncharted (7).

Traditional approaches such as immunohistochemistry and bulk transcriptomics cannot capture dynamic, spatially resolved cell-cell interactions in a heterogeneous lesion. Single-cell transcriptomics has transformed this landscape by enabling discovery of cell subsets and their signaling repertoires at unprecedented resolution. Spatial transcriptomics adds anatomical context, revealing where interactions occur (8, 9). Landmark efforts have demonstrated the power of integrating multimodal, four-dimensional measurements to dissect the molecular logic of SCI responses (8). However, most studies remain anchored to two-dimensional sections. A genuine three-dimensional reconstruction of cell-cell communication spanning the entire lesion, with time as the fourth dimension, is exceedingly rare. To our knowledge, no previous work has constructed a four-dimensional spatial atlas tracking microglia–fibroblast interactions after SCI (10). Building such an atlas is a necessary step toward bridging cellular heterogeneity with tissue-level organization.

In this study, we integrated spatial transcriptomics, single-cell transcriptomics, bulk RNA sequencing, and microarray profiling across multiple post-injury time points. From these data we constructed a four-dimensional spatial atlas of the injured spinal cord that captures how microglia and fibroblasts communicate over time and across three-dimensional space. This atlas revealed a dominant mode of microglia-to-fibroblast signaling that emerges precisely at the peak phase of fibrotic scar consolidation. Building on this mechanistic insight, we derived a novel clinical classification and risk prediction model based on fibroblast differentiation-associated gene signatures, enabling prospective identification of patients at high risk for excessive fibrosis. Using this stratification, we evaluated a targeted biomaterial-based intervention in the high-risk setting. The treatment was associated with reduced pathological fibrosis and improved functional recovery. Together, these findings identify microglia-fibroblast communication as a central feature of spinal cord fibrotic scar formation. The four-dimensional spatial atlas of injured and normal spinal cord, together with the fibroblast differentiation state-based classification and risk prediction model, provides a clinically actionable framework and opens new avenues for scar-targeting therapies after SCI.

## 2 Materials and methods

### 2.1 Data sources

Single-cell RNA-sequencing (scRNA-seq) data of mouse SCI were retrieved from the Gene Expression Omnibus (GEO) under accession GSE162610 (7). Microarray data of rat SCI (GSE69334) were obtained from GEO (11). RNA-seq data of human peripheral blood mononuclear cells (PBMCs) from SCI patients, trauma controls and healthy controls were accessed via GSE151371 (12). All raw microarray data were processed using the affy package, followed by robust multi-array average background correction, probe-specific background adjustment and probe-set summarization. Batch effects were corrected with the sva package. Transcription factor lists were obtained from the Cistrome database (http://cistrome.org/db/), and hallmark pathways were downloaded from the Molecular Signatures Database (MSigDB v7.1, https://www.gsea-msigdb.org/gsea/msigdb).

### 2.2 Animals and spinal cord injury models

All experimental procedures were approved by the Animal Welfare Committees of Tongji University. Female C57BL/6 mice (8–9 weeks old, 18–20 g) were used for bulk RNA-seq and scRNA-seq. Under anesthesia, a T9 laminectomy was performed, followed by a crush injury using No. 5 Dumont forceps mounted on a stereotaxic apparatus, with compression maintained for 3 s. For sham controls, laminectomy alone was performed without spinal cord compression. For spatial transcriptomics, 8-week-old C57BL/6 mice underwent T10 laminectomy under aseptic conditions. A contusion injury was delivered using the Impactor Model-III device, positioned 12.5 mm above the exposed spinal cord, with the impactor tip remaining in contact for 3 s. Sham controls received laminectomy only. Successful injury was confirmed by spinal cord congestion, oedema and bilateral hindlimb paralysis. For microarray analyses and NT3-chitosan intervention experiments, adult female Wistar rats (200–250 g) underwent T7-T8 laminectomy followed by excision of a 0.5 cm spinal cord segment. After surgery, rats received manual bladder expression twice daily until spontaneous voiding recovered. Animals were housed individually on softened bedding and monitored daily for body weight, wound condition, hydration, autophagia, and signs of urinary tract infection. Supportive care was provided according to institutional animal welfare guidelines. All mouse and rat experiments in this study were performed in female animals to maintain consistency across long-term surgical, behavioral, and multi-omics experiments. Sex was not included as a biological variable in the current study design.

### 2.3 Behavioral analysis

Locomotor recovery was assessed weekly using the Basso Mouse Scale for open-field locomotion. Mice were placed in an open-field arena and observed for 4 min by two trained investigators blinded to the experimental groups. Hindlimb movement, trunk stability, stepping patterns and coordination were scored according to the Basso Mouse Scale criteria.

For the rat complete transection model used in the NT3-chitosan intervention experiment, hindlimb locomotor recovery was evaluated using the Basso, Beattie, and Bresnahan locomotor rating scale. BBB scoring was performed before surgery, at 1 day after surgery, and weekly thereafter by observers blinded to treatment allocation. Eight rats per group were included in the longitudinal behavioral analysis. At 52 weeks after surgery, four of the eight rats in each group were randomly selected for re-transection at the original lesion site to determine whether the recovered locomotor function depended on continuity of the reconstructed spinal cord tissue. BBB scoring was then continued for the subsequent 5 weeks.

### 2.4 Bulk RNA-seq and microarray profiling

For bulk RNA-seq, spinal cord tissues centered on the lesion were collected from uninjured mice (n = 6) and at multiple post-injury time points (15 min, n = 3; 1 d, n = 3; 3 d, n = 6; 7 d, n = 6; 14 d, n = 5; 28 d, n = 6; 42 d, n = 4). Total RNA was extracted with TRIzol and purified using the RNeasy Mini Kit. Libraries were prepared with the Illumina V2 RNA-seq kit and sequenced on a HiSeq 2000 platform. Raw reads were quality-controlled with FastQC, aligned to the mm10 genome using HISAT2 and quantified with featureCounts. Differential expression analysis was performed with DESeq2 using thresholds of |log_2_fold change (FC)| > 0.5 and false discovery rate (FDR) < 0.05.

For microarray profiling, 5 mm spinal cord segments from four rats were pooled per biological sample (n = 167 total samples). Samples were collected from uninjured controls (n = 3) and from rostral, middle and caudal regions at 1, 3, 10, 20, 30, 60 and 90 days post-injury. RNA was extracted as above and hybridized to Affymetrix GeneChip Rat Genome 230 2.0 arrays. Raw data were processed with the affy package and robust multi-array average normalization, with batch effects corrected using the sva package. Differential expression was analyzed with the limma package.

### 2.5 Integration of bulk and single-cell transcriptomics

To identify injury-associated microglial and fibroblast subpopulations, bulk RNA-seq and scRNA-seq data were integrated using the Scissor algorithm (13). Scissor quantifies the similarity between each single cell and each bulk sample, then selects cells whose aggregated expression correlates most strongly with a given phenotype. Here, bulk expression profiles from SCI and sham-operated animals were used as the phenotype-associated reference. For each cell type, cells were ranked by their correlation strength, and a threshold was applied to define injury-associated and injury-resistant subsets.

### 2.6 Single-cell RNA-seq processing and cell type annotation

Single-cell suspensions from 10 mm spinal cord segments were dissociated using papain (2 mg mL^−^¹ in Hibernate A-Ca) at 30 °C for 10 min, followed by mechanical trituration. Viability exceeded 85% by trypan blue exclusion. Libraries were constructed with the Chromium Single Cell 3′ Kit v3 (10x Genomics) targeting 10,000 cells per sample and sequenced on an Illumina NovaSeq 6000. Raw reads were aligned to the mm10 reference genome (Ensembl 84) and quantified with Cell Ranger (v3.1.0). Downstream analysis was performed with the Seurat package. Cells with <200 detected genes or >10% mitochondrial reads were excluded. Normalization used the LogNormalise method (scale factor 10,000). The top 5,000 variable genes were identified via FindVariableFeatures. Principal component analysis (PCA) was performed, and the first 20 principal components were used for uniform manifold approximation and projection visualization and graph-based clustering. Differential expression was assessed with FindAllMarkers (Wilcoxon rank-sum test, |log_2_FC| > 0.5, FDR < 0.05). Cell types were annotated using canonical markers from the CellMarker database (http://xteam.xbio.top/CellMarker/) together with established lineage-restricted biomarkers. In particular, resident microglia were annotated on the basis of microglia-enriched transcripts including *P2ry12*, *Tmem119*, *Cx3cr1*, and *Hexb*, whereas infiltrating macrophage/myeloid populations were distinguished by enrichment of inflammatory and monocyte-associated markers. Subpopulation analysis was performed using RunUMAP and FindAllMarkers to identify distinct cellular states within major cell types.

### 2.7 Trajectory analysis

Pseudo-temporal ordering of microglial and fibroblast cells was analyzed with the Monocle2 package using the top 1,500 variable genes (14). The reduceDimension function with the DDRTree method was used to construct single-cell trajectories. Cells were ordered along pseudotime, and branch expression analysis modelling was performed to identify genes with differential expression patterns along the trajectories. For microglia fate-related gene identification, 1,147 genes differentially expressed across pseudotime were intersected with genes identified by univariate regression analysis of the microarray dataset to yield 36 differentially expressed microglia fate-related genes. A similar approach was applied to fibroblast populations to identify fibroblast differentiation-associated gene signatures.

### 2.8 Cell-cell communication analysis

Cellular communication networks were analyzed using CellChat and iTALK. For iTALK analysis, mouse genes were homologously mapped to human orthologs using the biomaRt package with the getLDS function. The normalized expression matrix of the top 1,500 variable genes served as input. Significant interactions were identified via permutation test, and the top 200 ligand-receptor pairs were visualized using ligand-receptor plots and iTALK networks. For NicheNet analysis, a sender-agnostic approach was adopted to predict active ligand-target links between microglia and fibroblasts (15). The nichenetr package was used to infer ligand-receptor interactions that potentially regulate gene expression changes in receiver cell populations, with a focus on day 7 post-injury as the peak phase of fibrotic scar consolidation.

### 2.9 Spatial transcriptomics and Bayesian niche identification

Spinal cord samples from sham (n = 3), 7 dpi (n = 4) and 14 dpi (n = 4) mice were embedded in optimal cutting temperature compound. Ten-micrometer cryosections were mounted on Visium Spatial Gene Expression slides (10x Genomics). Tissue permeabilization was optimized using the Visium Spatial Tissue Optimization Kit, with 6 min for sham and 12 min for injured tissues selected as optimal. Libraries were constructed according to the Visium Spatial Gene Expression user guide and sequenced on an Illumina NextSeq 550. Sequencing data were processed with Space Ranger (v1.0.0) for alignment to the GRCh38 genome and spot-by-gene matrix generation. Quality control removed spots with < 100,000 total transcripts or > 10% mitochondrial reads. Data integration across samples was performed with the IntegrateData function in Seurat. Cell type deconvolution of spatial transcriptomics data was performed using SPOTlight. To identify spatially distinct transcriptional domains within the lesion microenvironment, BayesSpace was applied (16). This fully Bayesian statistical method uses information from spatial neighborhoods for clustering analysis of spatial transcriptomic data and enhances resolution to identify finer spatial structures within the fibrotic niche. Spots were clustered into spatial domains using default parameters, and the resulting clusters were annotated based on known lesion compartment markers. The spatial niche corresponding to the fibrotic scar was identified based on enrichment of fibroblast-associated gene signatures and extracellular matrix components.

### 2.10 Four-dimensional spatial atlas of the injured spinal cord

To construct a four-dimensional spatial atlas capturing the spatiotemporal evolution of cell-cell communication after SCI, the publicly available spatial transcriptomics datasets from the *Tabulae Paralytica* resource were used (8). This resource comprises two-dimensional and three-dimensional spatial transcriptomics atlases of the injured mouse spinal cord, spanning four spatial and temporal dimensions. The three-dimensional spatial atlas was constructed by collecting 16 tissue sections equally spaced along the dorsoventral axis (approximately 50 µm apart) from uninjured mice and at 7 dpi and 2 months post-injury. All sections were registered to a common three-dimensional coordinate system. The four-dimensional atlas integrates the two-dimensional and three-dimensional spatial datasets with temporal information across multiple post-injury time points. To map single-nucleus transcriptomic data onto the spatial atlas, cell type deconvolution and spatial mapping were performed using RCTD as previously described. This integrated framework allowed visualization of the spatial distribution of microglia-fibroblast ligand-receptor interactions across the entire lesion in three dimensions and over time.

### 2.11 Construction of fibroblast differentiation-based molecular classification and risk prediction model

A novel molecular stratification of SCI patients was developed based on fibroblast differentiation-associated gene signatures. Briefly, fibroblast differentiation states were identified by integrating trajectory analysis and non-parametric tests on scRNA-seq data. Fibroblast differentiation-associated genes were defined as the intersection of genes differentially expressed across pseudotime. Consensus clustering (ConsensusClusterPlus) was performed on the expression profiles of these genes to identify optimal cluster numbers (17). To construct a risk prediction model, LASSO regression was applied to the training set (100 samples) to select the most informative genes. The model was validated in an independent test set (64 samples) and the entire SCI cohort.

### 2.12 External validation using public datasets

The robustness and clinical applicability of the fibroblast differentiation-based classification and risk prediction model were independently validated using two external public datasets. First, the microarray dataset GSE69334 (*Rattus norvegicus*) was used to validate the molecular subtypes and the prognostic value of the principal component analysis fibroblast score across different time points and treatment conditions. Second, the human PBMC RNA-seq dataset GSE151371 was used to validate the diagnostic potential of fibroblast differentiation-associated gene signatures detectable in peripheral blood. For this validation, consensus cluster-specific fibroblast differentiation-related genes were homologously mapped from rat to human using the biomaRt package and evaluated as surrogate transcriptional signatures in PBMCs. Differential expression analysis among SCI, trauma control and healthy control groups was performed, and genes that were significantly and specifically altered post-SCI (|log_2_FC| > 2, FDR < 0.05) were retained. Expression levels of these PBMC-detectable signatures were correlated with American Spinal Injury Association Impairment Scale grades using non-parametric tests to assess their ability to reflect injury severity and long-term outcomes.

### 2.13 Immunohistochemistry and image analysis

Tissue sections were blocked with 5% normal goat serum and 0.2% Triton X-100 for 1 h, then incubated with primary antibodies at 4 °C overnight. Primary antibodies used included chicken anti-Nestin (1:500), goat anti-Iba1 (1:800), rat anti-CD68 (1:500), rabbit anti-Pax6 (1:500), rabbit anti-GFAP (1:1000), rabbit anti-Col1a2 (1:500) and guinea pig anti-NeuN (1:500). After washing, sections were incubated with appropriate Alexa Fluor-conjugated secondary antibodies (1:1000) for 2 h at room temperature. Nuclei were counterstained with DAPI. Confocal z-stack images were acquired using a Zeiss LSM 880 microscope with a 20× objective (NA = 1.0) and analyzed using Imaris software for three-dimensional reconstruction and cell quantification.

### 2.14 Quantitative real-time PCR

Total RNA was extracted using TRIzol reagent and reverse-transcribed into cDNA. Quantitative PCR was performed with SYBR Green Master Mix on a QuantStudio 6 Flex system. Gene expression was quantified using mouse-specific primers. Relative gene expression of *IL-1α*, *IL-1β*, *NF-kB*, *IL-6*, and *TGF-β* was calculated using the 2−ΔΔCt method with β-actin as the endogenous control.

### 2.15 Statistical analysis

All statistical analyses were performed using R (v4.3.1), GraphPad Prism 9 or Python (v3.6). Data are presented as mean ± SEM. For comparisons between two groups, an unpaired two-tailed Student’s t-test was used. For multiple comparisons, one-way ANOVA followed by Bonferroni *post hoc* test was applied for single-factor analyses, and repeated-measures ANOVA followed by Bonferroni *post hoc* test was used for time-course behavioral data. A two-tailed *P*-value < 0.05 was considered statistically significant. All experiments were replicated with at least three biologically independent samples.

## 3 Results

### 3.1 A spatial transcriptomic atlas reveals time-dependent cellular niches and dynamic fibroblast states after SCI

**Figure 1:**
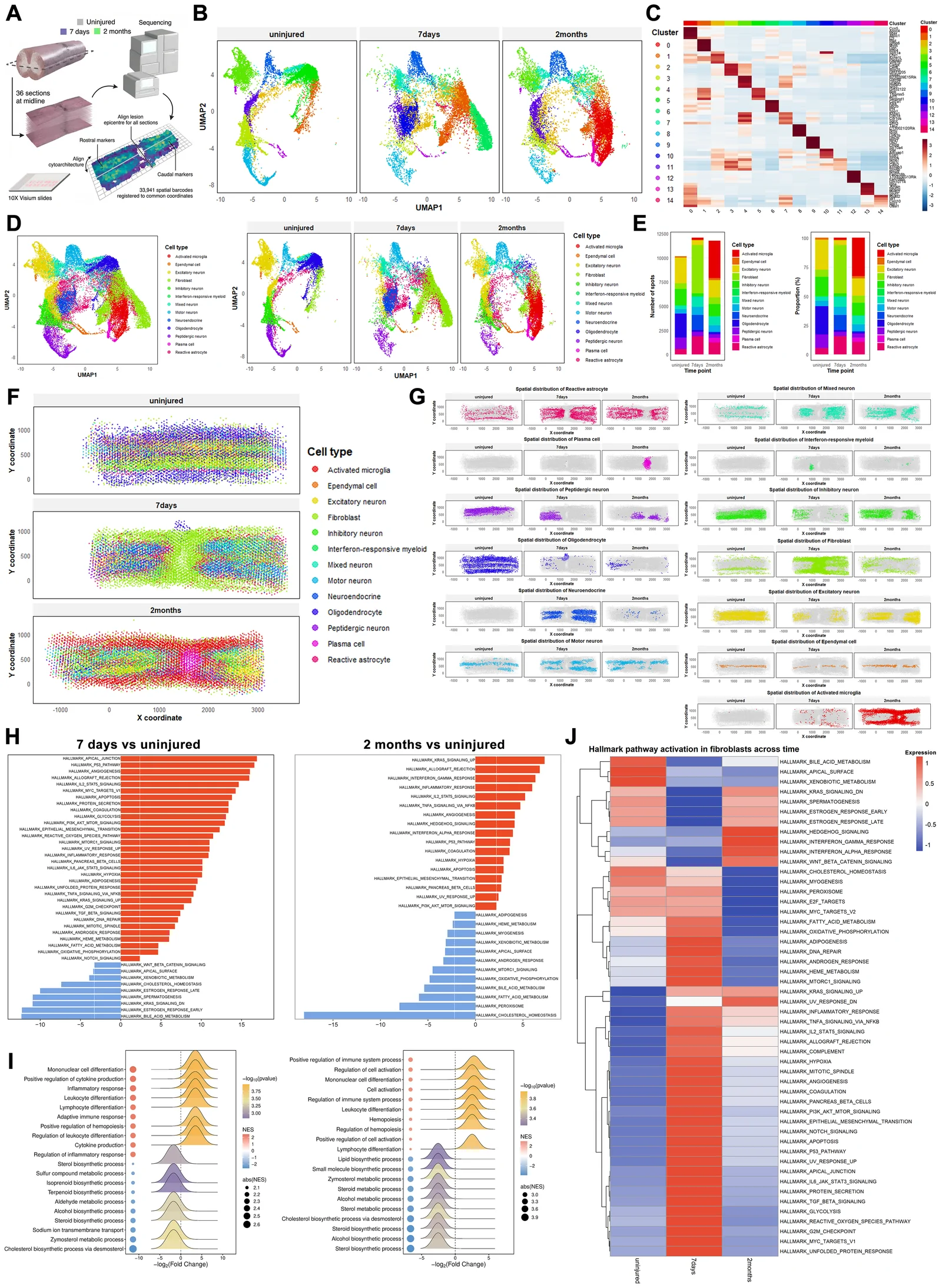
Spatial transcriptomic mapping of injury-induced cellular reorganization and dynamic fibroblast states. **(A)** Workflow of spatial transcriptomics sample preparation and analysis. **(B)** UMAP visualization of 33,941 spatial spots from uninjured spinal cord and lesions at 7 days and 2 months after SCI, colored by 15 transcriptionally distinct spatial states. **(C)** Dot plot showing expression of canonical marker genes used to annotate major neural, glial, stromal, and immune populations. **(D)** Spatial maps of cell type annotation in representative sections of uninjured, 7 dpi and 2 months samples. **(E)** Bar plot showing the fraction of each cell type per condition (uninjured, 7 dpi, 2 months). **(F)** Spatial distribution of major cell types in transverse sections at 7 dpi. **(G)** Spatial distribution of fibroblasts, reactive astrocytes and immune populations in vertical sections at 7 dpi. **(H)** Heatmap of GSVA enrichment scores for selected pathways in fibroblasts across uninjured, 7 dpi and 2 months. **(I)** GSEA enrichment plots for inflammatory and cytokine-associated processes in fibroblasts at 7 dpi versus uninjured. **(J)** Hallmark activity maps showing dynamic reconfiguration of fibroblast signaling pathways over time.

We generated a spatial transcriptomic atlas of the injured spinal cord by profiling uninjured tissue together with lesions at 7 days and 2 months after SCI, yielding 33,941 high-quality spatial barcodes from 36 transverse sections registered to a common anatomical framework. This design enabled direct comparison of injury-responsive programs across time within preserved tissue architecture and provided a spatially resolved view of how the lesion microenvironment evolves from the acute to chronic phase (**Figure 1A**). Unsupervised clustering resolved 15 transcriptionally distinct spatial states, which remained reproducible across conditions yet shifted markedly after injury, indicating broad remodeling of the cellular landscape over time (**Figure 1B**). Marker-based annotation further assigned these states to major neural, glial, stromal, and immune populations, including fibroblasts, reactive astrocytes, activated microglia, interferon-responsive myeloid cells, and multiple neuronal classes, thereby establishing the cellular framework for spatial analysis (**Figures 1C, D**).

Quantification of spot composition revealed a pronounced injury-induced redistribution of cell types, with expansion of stromal and immune compartments and a relative depletion of neuronal and oligodendroglial signatures, most prominently at 7 days and only partially resolved by 2 months (**Figure 1E**). Spatial mapping localized these changes to discrete anatomical territories and showed that the post-injury cord is organized by highly structured, time-dependent cellular niches rather than diffuse tissue-wide perturbation (**Figure 1F**). When resolved by individual cell type, fibroblasts and reactive astrocytes accumulated around the lesion domain, whereas immune populations concentrated within and adjacent to the injured core, consistent with progressive establishment and subsequent remodeling of the scar-associated microenvironment (**Figure 1G**).

Within fibroblasts, pathway analysis revealed a sharp temporal transition in functional state. At 7 days, GSVA identified enrichment of programs linked to apical junction organization, p53 signaling, and angiogenesis, consistent with rapid matrix remodeling, stress adaptation, and vascular niche formation. By 2 months, this profile shifted, indicating that fibroblasts move away from an acute injury-response state toward a more persistent remodeling program (**Figure 1H**). GSEA reinforced this pattern, with early enrichment of inflammatory and cytokine-associated biological processes, including mononuclear cell differentiation, positive regulation of cytokine production, inflammatory response, and positive regulation of immune system process, whereas chronic-stage fibroblasts retained a more selective and attenuated immune-remodeling signature relative to uninjured tissue (**Figure 1I**). Concordantly, Hallmark activity maps showed dynamic reconfiguration of fibroblast signaling across time, including prominent changes in WNT and β-catenin signaling, inflammatory response, and angiogenesis, supporting the view that fibroblasts are not merely structural components of the scar but active organizers of the evolving lesion ecosystem across distinct phases of SCI repair (**Figure 1J**).

### 3.2 Stage-specific rewiring of immune–stromal communication after

CellChat analysis revealed that spinal cord injury reshaped intercellular communication into a highly organized, injury-centered network dominated by glial, stromal, and myeloid populations rather than neuronal compartments. At 7 dpi, the global interaction map showed dense communication among activated microglia, fibroblasts, reactive astrocytes, and interferon-responsive myeloid cells, indicating rapid assembly of a coordinated inflammatory and reparative niche within the lesion environment (**Supplementary Figure 1A**). This architecture was not diffuse. It was sharply concentrated in cell populations that are known to define the evolving scar and immune microenvironment.

Quantification of interaction number further supported this hierarchy and identified activated microglia and fibroblasts as major communication hubs in the acute phase, both as senders and as receivers. Their prominence, together with substantial participation of reactive astrocytes and interferon-responsive myeloid cells, suggests that early post-injury signaling is organized around reciprocal immune stromal coupling rather than neuron-centered communication, consistent with active lesion remodeling and rapid niche specification after injury (**Supplementary Figure 1B**).

At higher resolution, ligand-receptor analysis highlighted a selective signaling architecture centered on activated microglia and fibroblasts. Among these interactions, *Spp1* to *Itgav*/*Itgb1* emerged as a particularly notable axis linking activated microglia with fibroblasts, placing osteopontin-integrin signaling at the interface between inflammation and matrix remodeling. Additional signals, including *Fn1* to *Cd44*, *Fn1* to *Itgav*/*Itgb1*, *Psap* to *Gpr37*, and *Ptn* to *Ncl*, further suggest convergence on extracellular matrix engagement, trophic support, and stress adaptation within the acute lesion niche (**Supplementary Figure 1C**). Complementary analysis from the receiver perspective showed that activated microglia and fibroblasts also integrate diverse incoming cues, including *Sema4d* to *Plxnb2*, *Col1a1* to Itgav/*Itgb8*, and *Fgf1* to *Fgfr2*, consistent with bidirectional information flow that couples inflammatory activation to stromal organization (**Supplementary Figure 1D**).

By 2 months post-SCI, the communication network remained robust but was qualitatively reconfigured, indicating transition from an acutely inflammatory state to a more consolidated chronic lesion ecosystem. The global network still centered on activated microglia and fibroblasts, yet the overall pattern appeared less explosively distributed and more structurally entrenched, suggesting that chronic communication is maintained through persistent scar-associated circuits rather than broad early injury signaling (**Supplementary Figure 1E**). This interpretation was reinforced by the interaction heatmap, which showed a redistributed communication burden across sender and receiver populations, consistent with maturation of the lesion microenvironment and selective retention of chronic signaling routes (**Supplementary Figure 1F**).

This temporal shift was also evident at the ligand-receptor level. At 2 months, outgoing communication from activated microglia and fibroblasts was characterized by pathways such as *C3* to *Itgax*/*Itgb2*, *App* to *Cd74*, and *Mif* to *Cd74*/*Cd44*, pointing to sustained innate immune surveillance, antigen-associated signaling, and chronic inflammatory reinforcement rather than the broader matrix-activating profile seen earlier (**Supplementary Figure 1G**). Incoming signaling to activated microglia and fibroblasts remained enriched for *Col1a1* to *Sdc4*, *Sema4d* to *Plxnb2*, and *Fgf1* to *Fgfr2*, indicating that chronic scar tissue continues to be shaped by adhesive, guidance-related, and trophic signals, but within a more restricted and stabilized communication framework (**Supplementary Figure 1H**). Taken together, these data support a model in which cell-cell communication after SCI undergoes a phase transition from early inflammatory matrix-coupled reciprocity to chronic scar maintenance, with the activated microglia-fibroblast axis occupying a central position throughout. Within this framework, *Spp1* to *Itgav*/*Itgb1* likely marks a critical early instructive signal that helps establish the long-lived stromal immune interface of the injured cord.

### 3.3 A 4D spatial atlas defines lesion organization and microglia–fibroblast communication after SCI

**Figure 2:**
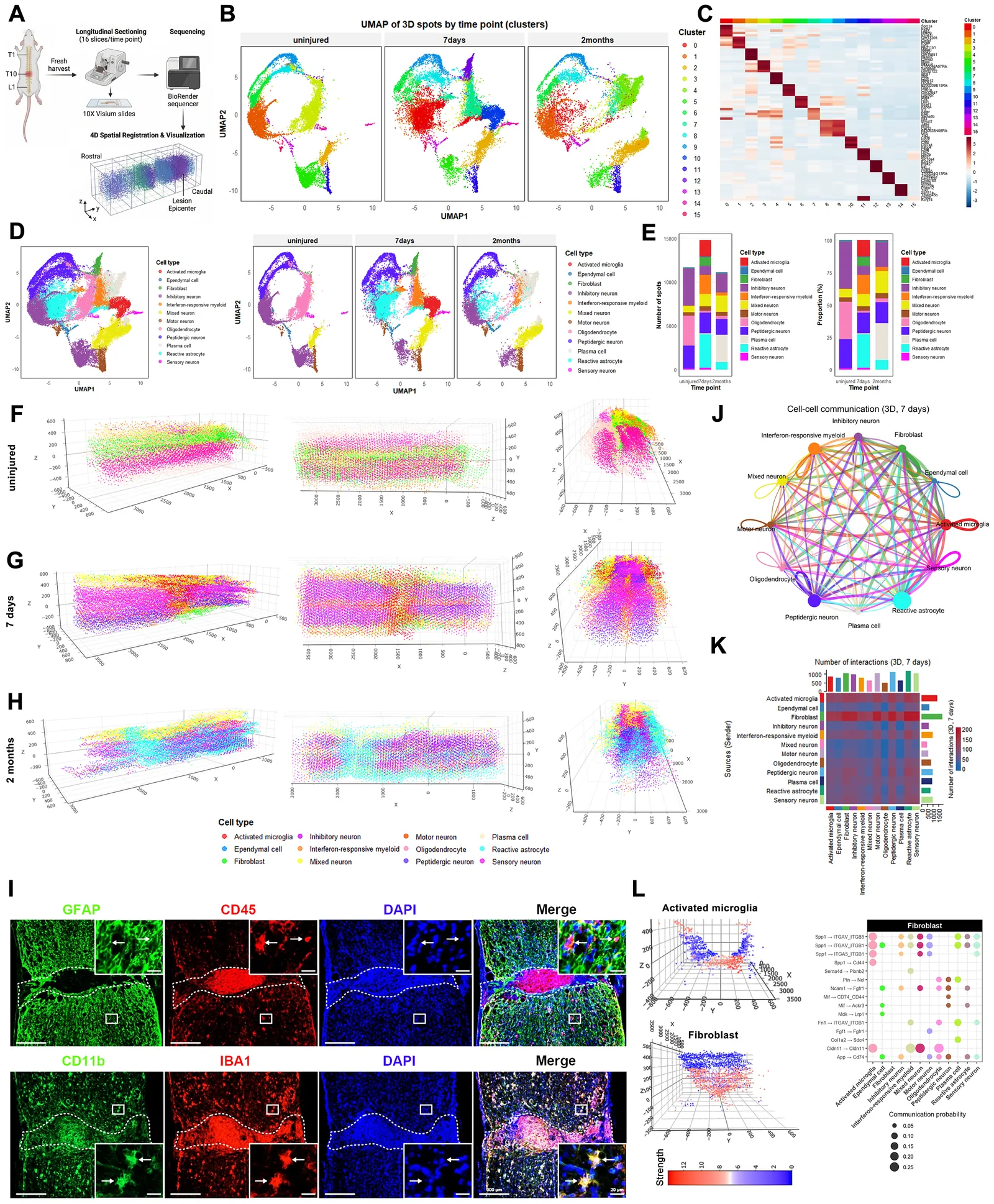
Four-dimensional reconstruction of lesion architecture and microglia-fibroblast communication. **(A)** Schematic of 4D spatial atlas construction using serial longitudinal sections registered to a common coordinate system. **(B)** UMAP of spatial spots colored by time after injury (uninjured, 7 dpi, 2 months). **(C)** Spatial maps of cell type annotation in 3D reconstruction at 7 dpi. **(D)** Bar plot of cell type abundance in uninjured, 7 dpi and 2 months. **(E)** 3D reconstruction of the uninjured spinal cord showing cell type distribution. **(F)** 3D reconstruction at 7 dpi showing collapse of tissue architecture and formation of a dense multicellular lesion core. **(G)** 3D reconstruction at 2 months showing compartmentalized lesion organization. **(H)** Immunofluorescence validation of astroglial (GFAP) and immune (CD45, CD11b, IBA1) compartments. **(I)** Three-dimensional CellChat network at 7 dpi highlighting activated microglia and fibroblasts as central communication hubs. **(J)** Quantification of incoming and outgoing interaction strength for each cell type. **(K)** Heatmap showing the number of interactions between different cell types. **(L)** Spatial visualization of Spp1–Itgav/Itgb1 ligand–receptor co-expression in the lesion core.

To resolve spinal cord injury as a spatiotemporally organized process, we generated a four-dimensional spatial atlas spanning the rostral to caudal axis, the dorsoventral extent, and successive post-injury stages. Serial longitudinal sampling followed by three-dimensional registration enabled reconstruction of the lesional territory in anatomical continuity, providing a framework to interpret cellular remodeling *in situ* rather than from isolated sections alone (**Figure 2A**).

Unsupervised analysis separated spatial spots into discrete states that were strongly structured by time after injury. Uninjured tissue occupied a compact transcriptional space, whereas samples from 7 days and 2 months after SCI shifted into distinct manifolds, indicating that acute injury and chronic remodeling represent biologically separate tissue states rather than different degrees of the same response (**Figure 2B**). Marker genes further resolved these states into activated microglia, interferon responsive myeloid cells, reactive astrocytes, fibroblasts, oligodendrocytes, and multiple neuronal populations, allowing inflammatory, stromal, and neural compartments to be mapped within a unified spatial framework (**Figure 2C**). Cell type annotation (**Figure 2D**) and abundance analysis (**Figure 2E**) showed a marked expansion of activated microglia, fibroblasts, and reactive astrocytes after injury, with persistent overrepresentation at the chronic stage. This pattern suggests that SCI evolves into a stable immune stromal niche rather than returning toward the pre injury baseline.

Three-dimensional reconstruction clarified how this transition is organized in space. In the uninjured cord, cell types remained orderly and spatially segregated across viewing angles, consistent with preserved tissue architecture and intact dorsoventral patterning (**Figure 2F**). By 7 days after injury, this organization collapsed around the lesion epicenter and was replaced by a dense multicellular core enriched for activated microglia, fibroblasts, and other non-neuronal populations that extended across multiple planes. Different angular views showed that this response was not confined to a single section, but formed a continuous volumetric lesion domain with convergent immune and stromal accumulation along the injured axis (**Figure 2G**). At 2 months, the lesion adopted a more consolidated but still abnormal spatial configuration. Reactive astrocytes and plasma cells occupied the lesion center, whereas activated microglia and fibroblasts remained enriched in the adjacent lesion associated domain, suggesting that chronic SCI evolves toward a compartmentalized rather than resolved tissue state (**Figure 2H**).

Immunofluorescence supported these spatial assignments and confirmed the close juxtaposition of astroglial and immune compartments within injured tissue. GFAP-positive borders, CD45-positive infiltrates, and CD11b/IBA1-positive lesion-associated myeloid cells recapitulated the cellular geography inferred from the atlas, supporting the fidelity of the reconstructed 4D map (**Figure 2I**). Because these immunolabels are not specific for resident microglia, microglial identity in the atlas was defined primarily by transcriptomic annotation rather than immunostaining alone.

Three-dimensional CellChat analysis at 7 days identified activated microglia and fibroblasts as prominent communication hubs embedded within a broader network of glial, stromal, immune, and neuronal interactions (**Figure 2J**). Quantification of inferred interactions showed that both populations contributed disproportionately as senders and receivers, placing them at the center of lesion sensing and structural remodeling (**Figure 2K**). Direct spatial visualization of their communication highlighted a strong enrichment of adhesion and matrix associated signaling, with Spp1 and Itgav/Itgb1 emerging as a particularly important axis within their shared lesional territory. In this setting, activated microglia appear positioned to engage integrin expressing fibroblasts at sites of close spatial apposition, thereby coupling innate immune activation to fibroblast retention, matrix organization, and scar consolidation. Additional pathways, including *Fn1* integrin and *Mif*, likely reinforce this circuitry, but the recurrent prominence of the *Spp1* integrin module places it near the core of the immune stromal interface that shapes lesion maturation in four dimensions (**Figure 2L**).

### 3.4 Spatial reorganization of the spinal cord lesion ecosystem after SCI

**Figure 3:**
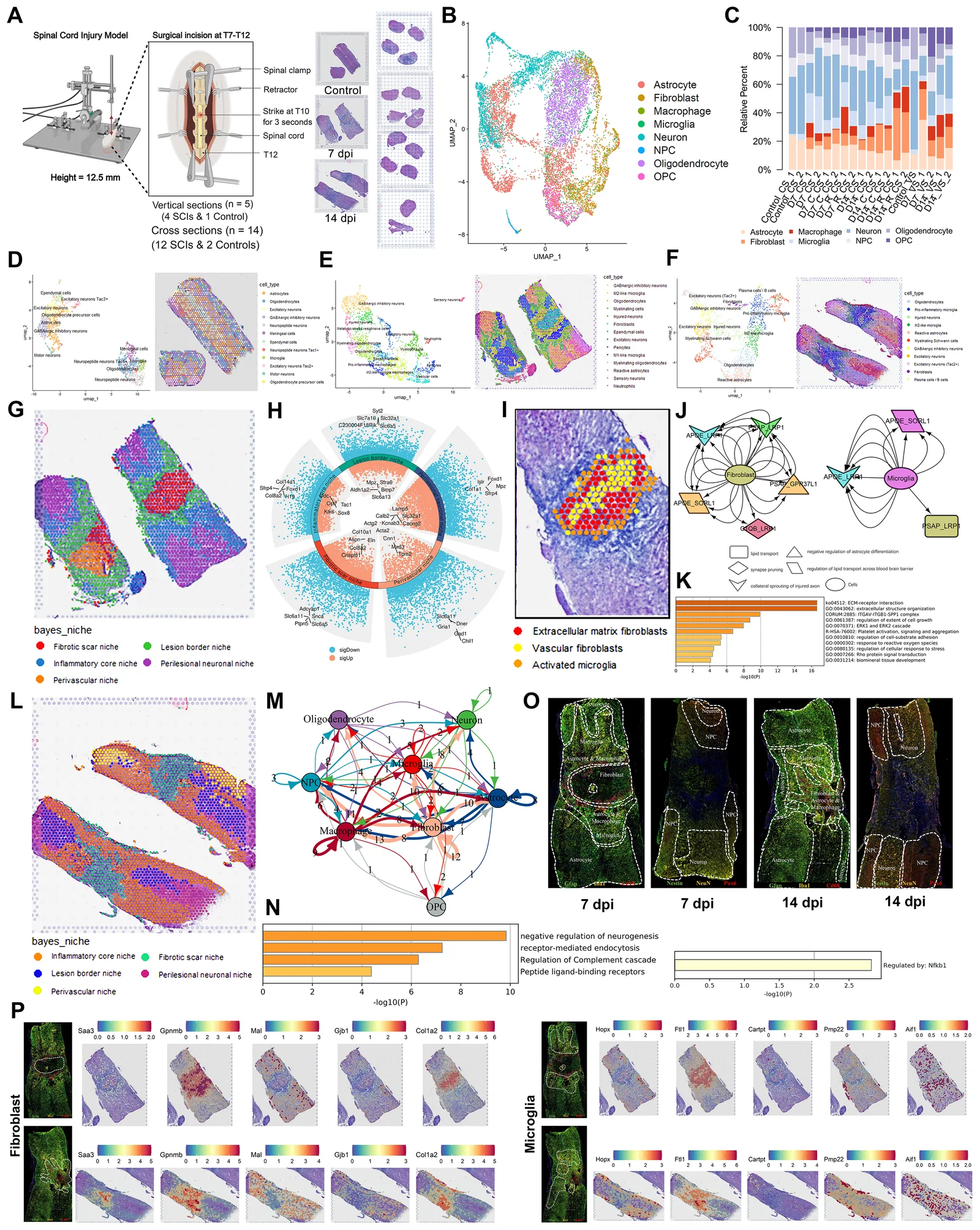
Temporal evolution of spatial niche segregation and sustained microglia-fibroblast crosstalk. **(A)** Schematic of in-house spatial transcriptomics sampling from uninjured, 7 dpi and 14 dpi mice. **(B)** UMAP of spatial spots colored by cell type. **(C)** Bar plot of cell type composition across conditions. **(D)** Spatial annotation of the uninjured cord. **(E)** Spatial annotation at 7 dpi. **(F)** Spatial annotation at 14 dpi. **(G)** Bayesian niche analysis at 7 dpi identifies five recurrent microenvironments: inflammatory core, fibrotic scar, lesion border, perilesional neuronal and perivascular niches. **(H)** Differential expression between fibrotic scar and inflammatory core niches. **(I)** Spatial localization of extracellular matrix fibroblasts, vascular fibroblasts and activated microglia. **(J)** Cell-cell communication network at 7 dpi. **(K)** Pathway enrichment of the Spp1–Itgav/Itgb1 axis. **(L)** Spatial niche mapping at 14 dpi. **(M)** Communication network at 14 dpi. **(N)** Functional analysis of the 14 dpi interactome. **(O)** Immunofluorescence validation of microglial and fibroblast compartments. **(P)** Spatial expression of fibroblast and microglial markers.

To define how SCI remodels tissue architecture over time, we generated in-house spatial transcriptomic maps from uninjured spinal cord and lesions collected at 7 and 14 days after injury across vertical and cross-sectional planes, establishing a temporally resolved framework for lesion analysis (**Figure 3A**). Unsupervised clustering identified the major neural, glial, stromal, and myeloid populations of the cord (**Figure 3B**), and compositional analysis showed that injury was accompanied by contraction of neuron- and oligodendrocyte-rich compartments together with expansion of fibroblast and injury-associated myeloid populations, consistent with progressive conversion of intact neural tissue into an immune-stromal lesion milieu (**Figure 3C**). Spatial annotation of the uninjured cord preserved the expected anatomical organization and served as the baseline reference state (**Figure 3D**). By 7 dpi, this organization was replaced by a sharply remodeled lesion landscape containing inflammatory, stromal, and injury-associated neural states, with activated microglial populations and fibroblast lineages already occupying distinct but adjacent territories within the damaged tissue (**Figure 3E**). At 14 dpi, these spatial domains became more consolidated, indicating transition from acute disruption to a stabilized pathological architecture rather than restoration of the pre-injury state (**Figure 3F**).

Bayesian niche analysis at 7 dpi resolved five recurrent microenvironments, including inflammatory core, fibrotic scar, lesion border, perilesional neuronal, and perivascular niches, that together structured the early lesion ecosystem (**Figure 3G**). Differential expression across these niches showed that the fibrotic scar was enriched for extracellular matrix and contractile programs, whereas the inflammatory core displayed a distinct injury-response signature, pointing to functional specialization embedded within spatial compartmentalization (**Figure 3H**). Consistent with this organization, extracellular matrix fibroblasts and vascular fibroblasts localized to scar-forming and perivascular territories, whereas activated microglia accumulated at the inflammatory interface where immune and stromal domains converged (**Figure 3I**). Cell-cell communication analysis at 7 dpi placed fibroblasts and microglia near the center of the lesion interactome and highlighted trophic, phagocytic, and lipid-associated signaling as dominant features of this crosstalk (**Figure 3J**). Pathway enrichment further emphasized extracellular matrix-receptor interaction, extracellular structure organization, and the ITGAV-ITGB1-SPP1 complex, supporting the idea that activated microglia engage scar-forming fibroblasts through an adhesive signaling program that is coherent with the Spp1-Itgav/Itgb1 axis defined in our preceding 4D atlas and communication analyses (**Figure 3K**). At 14 dpi, spatial niche mapping showed that the inflammatory core, fibrotic scar, lesion border, perilesional neuronal, and perivascular compartments remained clearly segregated, indicating persistence of lesion partitioning during chronic consolidation (**Figure 3L**). The inferred communication network at this stage remained strongly centered on microglia and fibroblasts, arguing that their interaction is sustained rather than transient during scar maturation (**Figure 3M**). Functional analysis of the 14 dpi interactome linked this network to negative regulation of neurogenesis, receptor-mediated endocytosis, complement-related processes, and *Nfkb1*-associated inflammatory control, placing microglia-fibroblast coupling upstream of both tissue remodeling and failed repair (**Figure 3N**). Independent immunofluorescence across 7 dpi and 14 dpi sections confirmed the spatial arrangement of major cell classes and further supported the progressive juxtaposition of microglial and fibroblast compartments within the evolving lesion (**Figure 3O**). Spatial expression of representative fibroblast and microglial markers across both 7 dpi and 14 dpi samples validated these assignments and reinforced the view that SCI reorganizes the cord into temporally stabilized pathological niches maintained by persistent microglia-fibroblast communication (**Figure 3P**).

### 3.5 Spatially organized microglia–fibroblast crosstalk is sustained by NF-κB and TGF-β transcriptional programs after SCI

**Figure 4:**
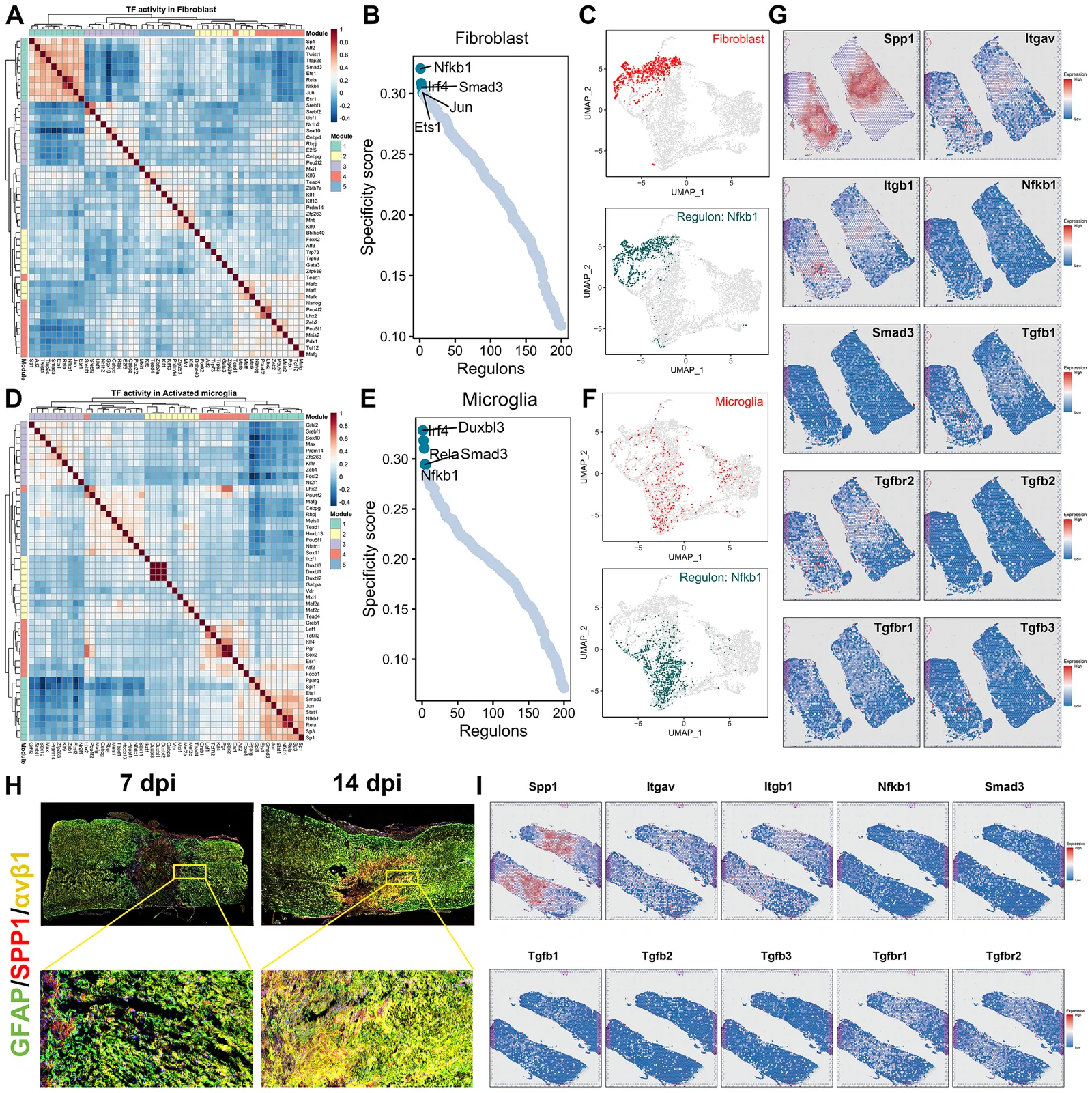
Convergent NF-κB and TGF-β regulons underpin spatially organized microglia-fibroblast crosstalk. **(A)** Regulon activity heatmap in fibroblasts at 7 dpi by SCENIC. **(B)** Regulon specificity analysis identifies *Nfkb1*, *Irf4*, *Smad3*, *Jun* and *Ets1* as fibroblast-associated regulators. **(C)** Mapping of the *Nfkb1* regulon onto the single-cell manifold shows enrichment in fibroblasts. **(D)** Regulon activity heatmap in activated microglia at 7 dpi. **(E)** Regulon specificity analysis in microglia. **(F)** Enrichment of the *Nfkb1* regulon in the microglial compartment. **(G)** Spatial co-expression of *Spp1*, *Itgav*, *Itgb1*, *Nfkb1*, *Smad3*, and TGF-β pathway genes at 7 dpi. **(H)** Immunofluorescence showing persistent apposition of SPP1 and integrin αvβ1 signals with GFAP-positive reactive tissue at 7 dpi and 14 dpi. **(I)** Spatial expression of *Spp1*, *Itgav*, *Itgb1*, *Nfkb1*, *Smad3*, and TGF-β-associated genes at 14 dpi.

Building on our 4D spatial atlas and cell–cell communication analyses, which identified activated microglia and fibroblasts as a major interacting pair linked by *Spp1*–*Itgav*/*Itgb1* signaling after SCI, we next used SCENIC to define the transcriptional programs underlying this spatially organized crosstalk. At 7 dpi, fibroblasts displayed a structured regulon landscape enriched for *Nfkb1*, *Smad3*, *Jun*, and *Ets1*, consistent with an injury-responsive state that couples inflammatory sensing to matrix remodeling rather than serving as a passive stromal scaffold (**Figure 4A**). Regulon specificity analysis further sharpened this pattern and identified *Nfkb1*, *Irf4*, *Smad3*, *Jun*, and *Ets1* as the most selective fibroblast-associated regulators, pointing to a convergent NF-κB- and TGF-β-linked transcriptional architecture within the fibrotic niche (**Figure 4B**). Mapping of the *Nfkb1* regulon onto the single-cell manifold showed that this activity was concentrated within fibroblasts, supporting the view that *NF-κB* signaling is intrinsic to the lesion-associated fibroblast state (**Figure 4C**). Activated microglia at the same stage exhibited a parallel but distinct regulatory program, with prominent activity of *Nfkb1*, *Rela*, *Smad3*, *Jun*, and *Stat1*, consistent with a lesion-reactive state integrating inflammatory activation with local environmental cues (**Figure 4D**). Specificity analysis again prioritized *Nfkb1* and *Smad3* together with *Rela* and *Irf4*, indicating that the two interacting populations share a core regulatory logic despite their different cellular identities (**Figure 4E**). The *Nfkb1* regulon was likewise enriched within the microglial compartment, arguing that NF-κB activation is a defining feature of activated microglia during the early post-injury phase rather than a diffuse consequence of tissue damage (**Figure 4F**).

These regulatory states were spatially anchored at 7 dpi by overlapping lesion-associated expression of *Spp1*, *Itgav*, *Itgb1*, *Nfkb1*, *Smad3*, and multiple TGF-β pathway components, including *Tgfb1*, *Tgfb2*, *Tgfb3*, *Tgfbr1*, and *Tgfbr2*, placing the previously inferred *Spp1*–*Itgav*/*Itgb1* interaction within an inflammatory and profibrotic signaling territory (**Figure 4G**). Immunofluorescence provided orthogonal validation *in situ* and showed persistent apposition of SPP1 and αvβ1 signals with GFAP-positive reactive tissue at both 7 dpi and 14 dpi, suggesting that this interface is maintained as the scar matures rather than being restricted to an early transient phase (**Figure 4H**). Consistent with that interpretation, spatial mapping at 14 dpi retained robust regional expression of *Spp1*, *Itgav*, *Itgb1*, *Nfkb1*, *Smad3*, and TGF-β-associated genes, indicating that microglia–fibroblast coupling is sustained during chronic lesion organization and likely reinforces fibrotic stabilization, inflammatory persistence, and the limited regenerative capacity of the injured spinal cord (**Figure 4I**).

### 3.6 Microglia--fibroblast signaling is associated with fibrotic scar maturation after SCI

**Figure 5:**
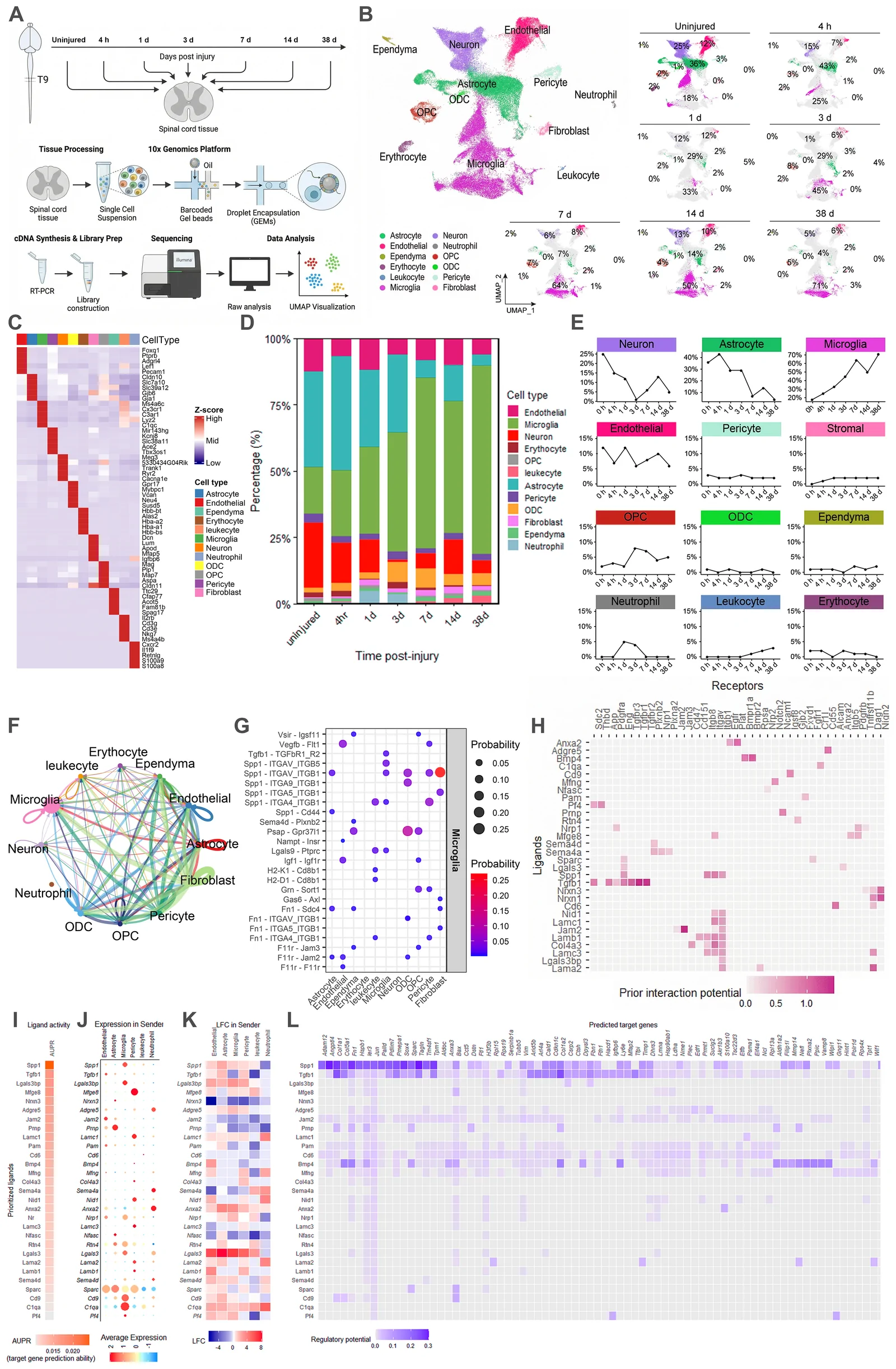
Single-cell transcriptomics identifies microglia-derived Spp1 as a driver of fibroblast profibrotic programs. **(A)** Schematic of in-house scRNA-seq sampling across acute and chronic stages. **(B)** UMAP of 12 major cell populations. **(C)** Dot plot of canonical lineage markers. **(D)** Relative abundance of major cell populations over time. **(E)** Temporal dynamics of neurons, astrocytes, microglia, fibroblasts, neutrophils and leukocytes. **(F)** CellChat network showing microglia as a central signaling hub. **(G)** Ligand-receptor dot plot highlighting Spp1–Itgav/Itgb1 as a top enriched pair. **(H)** Scissor-identified injury-associated microglia and fibroblast states. **(I)** NicheNet ligand activity ranking places Spp1 among top candidates. **(J)** Sender-cell expression of prioritized ligands. **(K)** Ligand induction analysis showing Spp1 upregulation in injury-associated sender states. **(L)** Predicted downstream targets in fibroblasts enriched for extracellular matrix, adhesion and vascular remodeling programs.

To resolve the cellular programs that stabilize the post-injury scar niche, we generated an in-house scRNA-seq dataset of injured spinal cord tissue spanning acute and chronic stages after SCI (**Figure 5A**). Unsupervised analysis identified 12 major cell populations encompassing neural, vascular, immune, and stromal compartments, revealing broad cellular diversification within the injured spinal cord (**Figure 5B**). Canonical lineage markers robustly validated cell annotation and established a high-confidence transcriptional atlas for downstream analysis of injury-associated cell-state transitions (**Figure 5C**). The relative abundance of major cell populations shifted markedly over time, indicating that SCI drives stepwise reorganization of the tissue ecosystem rather than a static inflammatory response (**Figure 5D**). This temporal remodeling was characterized by progressive loss of neurons and astrocytes, sustained expansion of microglia, early emergence and persistence of fibroblast-rich stromal populations, transient neutrophil infiltration, and delayed leukocyte accumulation, together defining the transition from acute injury to chronic scar maturation (**Figure 5E**).

To determine how these changing populations are functionally coupled, we reconstructed intercellular communication networks using CellChat. This analysis revealed extensive rewiring after SCI and positioned microglia as a central signaling hub candidate within the lesion niche (**Figure 5F**). Among microglia-centered interactions, signaling toward fibroblasts was especially prominent. Notably, the *Spp1*–*Itgav*/*Itgb1* axis emerged as one of the most enriched and biologically compelling ligand–receptor pairs, suggesting that activated microglia are a major upstream source of adhesion-linked profibrotic cues acting on fibroblasts (**Figure 5G**).

To connect this communication axis to the injury phenotype, we applied Scissor to identify damage-associated microglia and fibroblast states and then used NicheNet to infer ligand–receptor coupling within this prioritized sender–receiver framework. These analyses defined a restricted interaction landscape enriched for high-confidence regulatory signals between activated microglia and fibroblasts (**Figure 5H**). Ligand activity ranking further prioritized a small set of upstream signals capable of explaining the fibroblast transcriptional response, with *Spp1* standing out among the top candidates together with *Tgfb1* and other injury-associated mediators (**Figure 5I**). Analysis of sender-cell expression showed that these prioritized ligands were enriched in microglia and selected lesion-associated populations, supporting the view that activated microglia serve as a principal signaling source within the evolving scar microenvironment (**Figure 5J**). Consistent with this interpretation, ligand induction analysis demonstrated that *Spp1* and related candidate signals were selectively upregulated in injury-associated sender states, further strengthening the case for microglia-derived instructive signaling after SCI (**Figure 5K**). Predicted downstream targets in fibroblasts were enriched for extracellular matrix organization, adhesion, and vascular remodeling programs, including *Adam12*, *Angpt4*, and *Col15a1*, indicating that microglia-derived signals are coupled to a stable profibrotic transcriptional program. Together, these data identified microglia–fibroblast crosstalk as a prominent feature of lesion maturation and support Spp1 signaling through the Itgav/Itgb1 receptor complex as a candidate mechanism linking immune activation to persistent stromal remodeling after SCI (**Figure 5L**).

### 3.7 Independent single-cell validation supports the microglia–fibroblast signaling axis in SCI

We reanalyzed the mouse SCI scRNA-seq dataset GSE162610 across uninjured spinal cord and early post-injury time points. Cells from different samples showed good integration in low-dimensional space, indicating limited batch-driven separation and permitting direct comparison of injury-induced remodeling across conditions (**Supplementary Figure 2A**). Unsupervised clustering resolved 23 transcriptionally distinct cell clusters, revealing substantial cellular diversity within the injured cord (**Supplementary Figure 2B**). This global structure suggested that SCI induces not only changes in cell abundance, but also marked diversification of cell states. Cluster-enriched genes further supported the biological distinctness of these populations. Canonical inflammatory, stromal, myelin-associated, and neuronal markers segregated sharply across clusters, indicating robust lineage assignment and injury-dependent transcriptional specialization (**Supplementary Figure 2C**). On the basis of established marker genes, these clusters were consolidated into 15 major cell types spanning neural, vascular, immune, and stromal compartments (**Supplementary Figure 2D**). This annotation established the cellular architecture required to interpret lesion-associated remodeling at system level. The corresponding marker heatmap confirmed clear molecular identities for each annotated population. Microglia, fibroblasts, oligodendrocytes, endothelial cells, astrocytes, and infiltrating immune cells each displayed coherent and largely non-overlapping transcriptional signatures (**Supplementary Figure 2E**). The microglial cluster was supported by expression of canonical microglia-associated markers including *Hexb, P2ry12*, and *Tmem119*, distinguishing it from infiltrating macrophage/myeloid populations. Cellular composition changed profoundly after SCI. Immune and stromal populations expanded across injured samples, whereas homeostatic neural populations became relatively depleted, consistent with progressive conversion of the spinal cord microenvironment into an inflammatory and fibrotic niche (**Supplementary Figure 2F**). This shift was reinforced by representative gene patterns across clusters and cell types. *Dcn* marked fibroblast-enriched populations, *Aif1* identified microglial and myeloid compartments, and *Spp1* was selectively elevated in injury-associated states, pointing to activation of a conserved lesion-response program linked to tissue remodeling (**Supplementary Figure 2G**).

Cell-cell interaction analysis further showed that this remodeling was not cell autonomous. Microglia occupied a central position in the communication network and displayed extensive interactions with macrophages, endothelial cells, and fibroblasts, consistent with a coordinating role in the injured tissue microenvironment (**Supplementary Figure 2H**). At ligand-receptor resolution, these interactions converged on a restricted set of recurrent signaling nodes. Notably, *Spp1* appeared prominently within the intercellular network, including links connecting microglia and fibroblasts, providing orthogonal support for the *Spp1*-centered communication axis and reinforcing the relevance of *Spp1*–*Itgav*/*Itgb1* signaling during scar formation (**Supplementary Figure 2I**).

To resolve the stromal compartment in greater depth, we reclustered fibroblasts and identified seven transcriptionally distinct fibroblast subpopulations (**Supplementary Figure 2J**). Their clear segregation suggested that scar-associated fibroblasts comprise multiple specialized states rather than a uniform cell pool. These subclusters were subsequently annotated as Fibroblast1 to Fibroblast7, providing a structured framework for tracking fibroblast diversification after injury (**Supplementary Figure 2K**). This organization enabled finer dissection of how stromal programs evolve during scar maturation. Differential expression analysis demonstrated pronounced molecular divergence among fibroblast subpopulations. Distinct subsets were enriched for extracellular matrix genes, contractile markers, immune-associated transcripts, or remodeling factors, including *Col3a1*, *Dcn*, *Sfrp4*, *Igfbp5*, *Acta2*, *Tagln*, *Apoe*, *C1qa*, and *C1qb* (**Supplementary Figure 2L**). The marker heatmap highlighted discrete stromal programs associated with matrix production, myofibroblast-like activation, inflammatory coupling, and tissue remodeling (**Supplementary Figure 2M**). These data indicate that fibroblast heterogeneity is a defining feature of the evolving fibrotic scar. The abundance of fibroblast subpopulations changed dynamically across uninjured and injured samples, indicating stage-dependent redistribution of distinct stromal states during SCI progression (**Supplementary Figure 2N**).

### 3.8 Establishment of a fibroblast differentiation-based molecular classification of SCI

Cell-cycle projection of fibroblasts from GSE162610 revealed marked differences in proliferative state across subsets, indicating that fibroblast heterogeneity after SCI is accompanied by unequal cell-cycle engagement rather than uniform quiescence or activation (**Supplementary Figure 3A**). G2M and S phase scores varied across clusters, identifying subsets with stronger cycling signatures and others with weaker proliferative activity, thus separating fibroblasts with distinct activation states (**Supplementary Figure 3B**).

The iTALK analysis revealed extensive ligand–receptor communication among fibroblast subsets, indicating that these populations are embedded within an active intercellular signaling network rather than existing as transcriptionally isolated states (**Supplementary Figure 3C**). Consistently, the interaction landscape was dominated by signaling related to extracellular matrix organization, adhesion, and tissue remodeling, indicating that fibroblast diversification is coordinated through shared but specialized communication programs during scar formation (**Supplementary Figure 3D**).

Monocle2 reconstruction resolved a branched fibroblast trajectory, indicating that scar-associated fibroblasts progress through a differentiation continuum that diverges into distinct terminal states during SCI (**Supplementary Figure 3E**). Pseudotime mapping confirmed an ordered temporal progression along this branched structure, supporting the stability of the inferred differentiation landscape (**Supplementary Figure 3F**). State annotation further partitioned the trajectory into discrete yet connected transcriptional states, providing a framework for fibroblast fate diversification (**Supplementary Figure 3G**). Genes associated with these states were enriched in inflammatory signaling, microglial response, axon guidance, neuronal projection, and myelination-related pathways, indicating that fibroblast differentiation is coordinated with both immune remodeling and neural repair programs in SCI (**Supplementary Figure 3H**). Branch-dependent analysis identified gene modules that changed before and after fate bifurcation, distinguishing early/intermediate fibroblasts from branch-restricted terminal populations (**Supplementary Figure 3I**). Correlation analysis further resolved coordinated gene modules associated with specific trajectory states, revealing structured transcriptional programs underlying fibroblast state stabilization and branch commitment (**Supplementary Figure 3J**).

Using trajectory-derived fibroblast differentiation-associated genes, we established a new SCI molecular classification grounded in fibroblast differentiation status. In GSE69334, consensus clustering identified three robust subclasses as the optimal solution (**Supplementary Figure 3K**). At k = 3, the consensus matrix showed high within-subtype concordance and clear between-subtype separation, supporting the robustness of this differentiation-based classifier (**Supplementary Figure 3L**). The same three-subtype structure was independently reproduced in GSE151371, where consensus clustering again supported k = 3 as the most stable solution (**Supplementary Figure 3M**). Consistently, the consensus matrix confirmed strong cluster stability and reproducible sample assignment, establishing a transferable fibroblast differentiation-based taxonomy of SCI across independent cohorts (**Supplementary Figure 3N**).

### 3.9 The fibroblast differentiation-based molecular classification captures clinical and biological heterogeneity in SCI

To translate the fibroblast differentiation trajectory inferred from scRNA-seq into a clinically relevant framework, we extracted fibroblast differentiation-associated genes from scRNA-seq dataset and applied consensus clustering in independent bulk cohorts. This identified three reproducible SCI subtypes associated with injury timing, treatment exposure, neurological severity and immune remodeling. To quantify fibroblast differentiation at the sample level, we further calculated a fibroblast score based on the aggregate expression of fibroblast differentiation-related genes.

The three fibroblast differentiation-based SCI subtypes differed significantly across post-injury time, treatment group, injury status and ASIA grade (all *P* < 0.05, **Supplementary Figure 4A**). Clinically, Cluster 1 was enriched in controls and neurologically less severe samples, consistent with a relatively mild injury state, whereas Clusters 2 and 3 were dominated by SCI cases. Among them, Cluster 3 showed greater enrichment for severe ASIA categories, while Cluster 2 contained a larger proportion of less advanced injured cases, indicating that the classification captures clinically meaningful differences in neurological impairment.

Differentiation-associated genes showed distinct subtype-specific patterns across ASIA strata. Notably, Itgav, which we previously identified as a key fibroblast communication receptor, was preferentially enriched in the clinically more severe subtype together with activated stromal, vascular and immune-response programs, linking fibroblast signaling to neurological status and suggesting that Itgav is a molecular correlate of adverse clinical stratification (**Supplementary Figure 4B**). This transcriptional program remained stably stratified across post-injury time points and anatomical regions in mouse SCI, supporting the spatiotemporal robustness of the subtype framework (**Supplementary Figure 4C**).

Fibroblast scores increased progressively from Cluster 1 to Cluster 3, with significant differences in all pairwise comparisons (all *P* < 0.001, **Supplementary Figure 4D**). This trend paralleled the clinical distribution across clusters, with the fibroblast-low state corresponding to milder neurological categories and the fibroblast-high state corresponding to more severe injury. *Itgav* showed the same directional pattern, supporting its relevance to both fibroblast activation and clinical severity.

Consistent with this, the fibroblast-high group showed increased expression of canonical stromal and injury-responsive genes, including *Col1a2*, *Dcn*, *Jun*, and *Cd68* (all *P* < 0.001), consistent with enhanced matrix remodeling and inflammatory activation (**Supplementary Figure 4E**).

In GSE69334, PCA separated the three subtypes into discrete transcriptomic groups, confirming reproducibility in an independent SCI microarray cohort (**Supplementary Figure 4F**). The same three-subtype architecture was reproduced in GSE151371 despite differences in species, tissue source and platform, supporting the cross-cohort stability and translational relevance of the model (**Supplementary Figure 4G**). Fibroblast-score stratification revealed temporal bias after SCI, with fibroblast-high samples enriched at early and intermediate stages, indicating that fibroblast activation is most prominent during the active remodeling phase after injury (**Supplementary Figure 4H**). Sankey analysis further showed non-random relationships among subtype, fibroblast-score state, treatment and time, supporting the biological organization and interpretability of the classification across multiple clinical dimensions (**Supplementary Figure 4I**).

Most differentiation-associated genes were expressed at the highest levels in Cluster 3, including *Itgav*, supporting a graded hierarchy of fibroblast activation across the three subtypes. Together with its concordant increase with fibroblast score and enrichment in clinically more severe groups, this identifies *Itgav* as a plausible molecular indicator of poor neurological status within the subtype framework (**Supplementary Figure 4J**). Immune infiltration differed markedly among subtypes, with Cluster 3 showing broader immune enrichment than Clusters 1 and 2, indicating that this classification captures a clinically meaningful stromal-immune axis in SCI (**Supplementary Figure 4K**).

### 3.10 PBMC-detectable fibroblast differentiation-associated transcriptional signatures predict severe neurological impairment

In mouse SCI, fibroblast differentiation-associated genes showed differential expression across time, treatment and anatomical location (**Supplementary Figure 5A**). The volcano plot showed that *Itgav* was significantly upregulated in SCI compared to uninjured samples, which ranked among the top activated fibrotic genes (**Supplementary Figure 5B**). In terms of the differentially expressed transcription factors, *Nfkb1* and *Smad3* were significantly upregulated in the middle lesion regions (**Supplementary Figure 5C**). These genes were enriched in biological processes associated with positive regulation of neuron death, microglial cell activation, and leukocyte activation involved in inflammatory response (**Supplementary Figure 5D**). NT3-chitosan treatment effectively reversed this dysregulation.

In peripheral blood from patients with SCI, PBMC-detectable fibroblast differentiation-associated gene signatures showed a clear separation between ASIA grade A and non-A samples (**Supplementary Figure 5E**). We constructed a prediction model for ASIA grade A using PBMC-detectable fibroblast differentiation-associated gene signatures via LASSO regression (**Supplementary Figure 5F**). Across the total, training and test sets, Itgav was consistently elevated in the high-risk group (all *P* < 0.001, **Supplementary Figure 5G**). The risk prediction model showed good discriminatory performance with AUCs of 0.883 in the total set, 0.939 in the training set, and 0.807 in the test set (**Supplementary Figure 5H**).

The risk score also effectively resolved distinct biological states. High-risk samples exhibited increased immune cell infiltration (**Supplementary Figure 5I**) and were enriched for pro-inflammatory signaling pathways (**Supplementary Figure 5J**), whereas low-risk samples were enriched for myelin sheath, neuron fate specification, positive regulation of angiogenesis, and neuron projection-related programs (**Supplementary Figure 5J**). Thus, PBMC-detectable fibroblast differentiation-associated signatures have considerable predictive value for neurological severity and support peripheral blood as an accessible surrogate of central nervous system injury-associated molecular states. These blood-based signatures are best interpreted as shared fibrosis-related inflammatory programs measurable in circulating immune cells.

### 3.11 NT3-chitosan promotes repair by suppressing the fibrotic niche signaling

**Figure 6:**
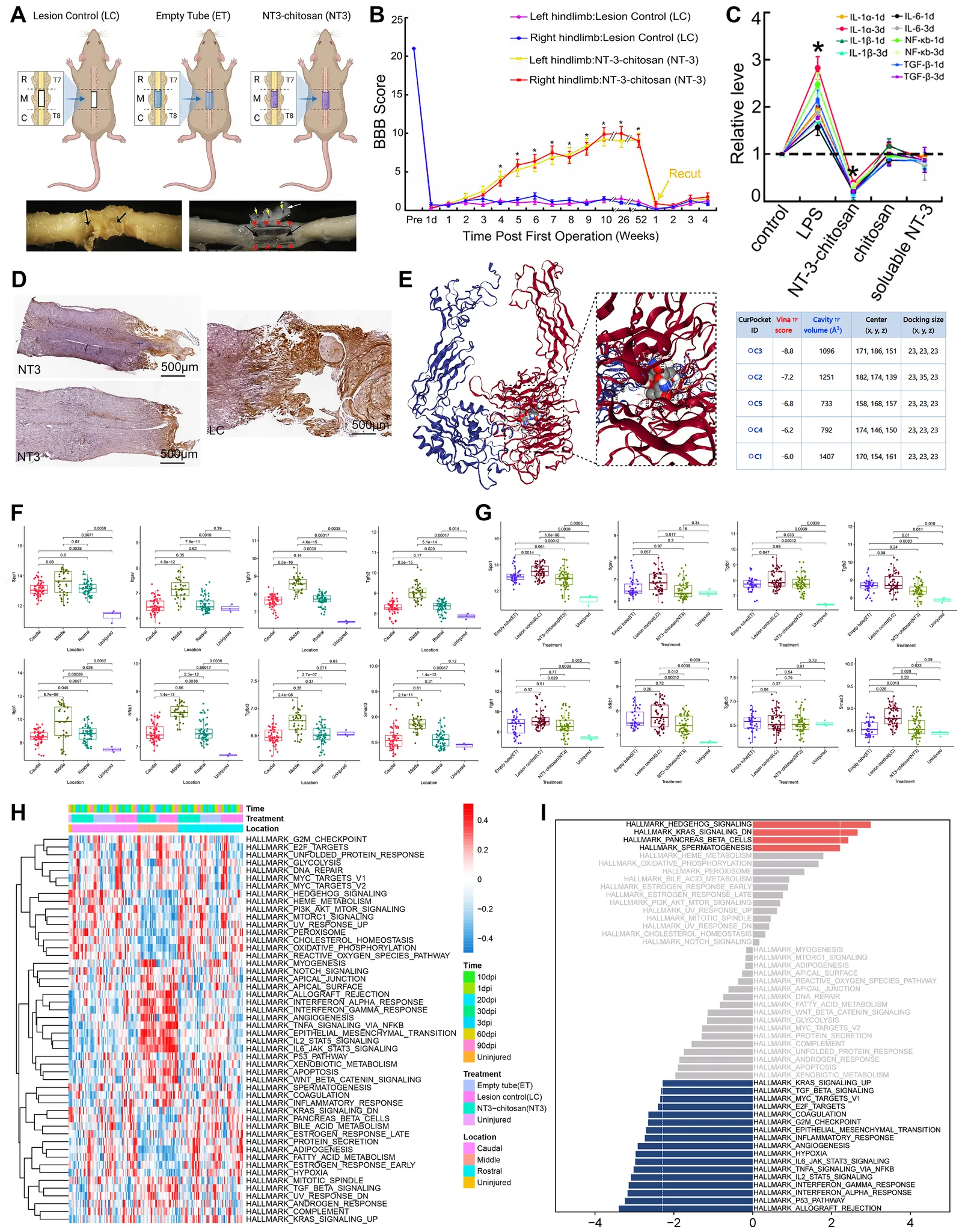
NT3-chitosan suppresses fibrotic niche signaling and promotes long-term functional recovery. **(A)** Gross anatomy of the T7-T8 complete transection model after treatment with NT3-chitosan, empty chitosan or lesion control. **(B)** BBB scores of the left and right hindlimbs in rats after complete thoracic spinal cord transection. NT3-chitosan-treated animals showed progressive functional recovery over 52 weeks, whereas lesion controls remained at low levels. At week 52, 4 of the 8 rats in each group underwent re-transection at the original lesion site, followed by 5 additional weeks of BBB scoring. In the NT3-chitosan group, BBB scores decreased from approximately 10 points before re-transection to near-baseline levels after re-transection, indicating that locomotor recovery depended on the continuity of the reconstructed spinal cord tissue. n = 8 per group. Mean ± SEM. Repeated-measures ANOVA with Bonferroni *post hoc* test. *P < 0.05 vs. lesion control. **(C)** Macrophage inflammatory cytokine expression (IL-1α, IL-1β, IL-6, NF-κB, TGF-β) after LPS stimulation with NT3-chitosan, chitosan alone, or soluble NT3. n = 4 per group. Mean ± SEM. One-way ANOVA with Bonferroni *post hoc* test. *P < 0.05 vs. control. **(D)** Representative histology showing tissue continuity and scar deposition. n = 3 per group. **(E)** Molecular docking of chitosan to the Itgav/Itgb1 complex. **(F)** Regional expression (rostral, middle, caudal) of Spp1, Itgav, Itgb1, Nfkb1, Tgfb1, Tgfb2, Tgfbr3 and Smad3. For each of the three regions (rostral, middle, caudal), n = 3 pooled samples per treatment group (lesion control, empty tube, NT3-chitosan) at each post-injury time point. Total samples: rostral n = 63, middle n = 38, caudal n = 63. Mean ± SEM. One-way ANOVA with Bonferroni *post hoc* test. **(G)** Expression levels of the same genes across treatment groups (lesion control, empty tube, NT3-chitosan). Sample sizes and statistical tests as in **(F)**. **(H)** GSEA of 50 hallmark pathways showing coordinated suppression of inflammatory, mesenchymal, hypoxic and angiogenic programs after NT3-chitosan. Sample sizes as in **(F)**. **(I)** GSVA showing reduced TGF-β signaling, inflammatory response, IL6-JAK-STAT3 signaling and TNFα signaling via NF-κB, and enriched hedgehog signaling. Sample sizes as in **(F)**.

Our previous work suggested that NT3-chitosan exerts a strong anti-inflammatory effect (11). We therefore next examined whether NT3-chitosan treatment was associated with changes in Spp1-Itgav/Itgb1-related crosstalk and downstream NF-kB- and TGF-β-associated signaling *in vivo* after complete thoracic spinal cord transection. In a T7 to T8 complete transection model with removal of a 5 mm spinal cord segment, the lesion cavity was left untreated, filled with an empty chitosan tube, or bridged with an NT3-loaded chitosan tube. Gross anatomical inspection and longitudinal follow-up showed stable integration of the NT3-chitosan graft and formation of bridging tissue across the lesion, in contrast to the persistent defect seen in lesion controls and the limited structural restoration in the empty-tube group, indicating that NT3-chitosan provides an anatomical substrate permissive for regeneration (**Figure 6A**). This tissue-level effect was accompanied by durable functional improvement. BBB scores progressively improved in the NT3-chitosan group over 52 weeks, whereas lesion controls showed persistently poor locomotor function. Re-transection of the repaired segment abolished this functional gain, reducing BBB scores from approximately 10 at 52 weeks to near-baseline levels, where they remained during the post-recut observation period with only slight recovery. This decline indicated that the recovered locomotion depended on the continuity of the reconstructed spinal cord tissue rather than compensatory adaptation alone (**Figure 6B**).

In macrophage-based inflammatory assays, LPS markedly increased the expression of pro-inflammatory and pro-fibrotic cytokines, including IL-1α, IL-1β, IL-6, NF-κB, and TGF-β, at both 1 and 3 days post-stimulation (all *P* < 0.05). Treatment with NT3-chitosan significantly suppressed the LPS-induced elevation of these transcripts (all *P* < 0.05), whereas chitosan alone or soluble NT3 showed much weaker effects (all *P* > 0.05) (**Figure 6C**). These data indicated that NT3-chitosan exerted a potent anti-inflammatory effect on activated macrophages. Histological analysis showed that NT3-chitosan-treated cords displayed improved tissue continuity and a marked reduction in scar-associated tissue relative to lesion controls (**Figure 6D**).

We then asked whether these phenotypic changes were accompanied by alterations in the Spp1-Itgav/Itgb1-associated receptor complex highlighted by the upstream cell communication analysis. Molecular docking suggested a potentially favorable interaction between chitosan and the Itgav/Itgb1 complex across several putative pockets, with the strongest predicted pose at pocket C3 achieving a Vina score of -8.8 (**Figure 6E**). While such in silico observations are hypothesis-generating rather than confirmatory, they provide a structural rationale for a potential interaction and warrant further validation by direct binding or competition assays.

Across anatomical regions, *Spp1*, *Itgav*, *Itgb1*, *Nfkb1*, and members of the TGF-β signaling pathway (*Tgfb1*, *Tgfb2*, *Tgfb3*, *Tgfbr3*, and *Smad3*) were preferentially elevated in middle lesion regions compared to rostral and caudal segments, whereas their expression remained low in uninjured tissue (**Figure 6F**). Among treatment groups, lesion control and empty-tube controls maintained persistently high expression of these genes. In contrast, NT3-chitosan treatment was associated with substantially reduced transcript levels, in some cases approaching uninjured baselines (**Figure 6G**).

GSEA of 50 hallmark pathways indicated coordinated suppression of inflammatory, mesenchymal, hypoxic, and angiogenic programs after NT3-chitosan treatment, consistent with destabilization of the chronic scar-forming milieu (**Figure 6H**). GSVA further identified reduced TGF-β signaling, inflammatory response, IL6-JAK-STAT3 signaling, and TNFα signaling via NF-κB, together with relative enrichment of hedgehog signaling, supporting a shift from a fibrotic inflammatory state toward a more permissive reparative environment (**Figure 6I**).

## 4 Discussion

Our study establishes microglia–fibroblast crosstalk as a spatiotemporally organized feature of fibrotic scar formation after spinal cord injury. By integrating single-cell, spatial and bulk transcriptomic platforms, we identified the Spp1–Itgav/Itgb1 signaling axis as a central communication node that links inflammatory activation to stromal remodeling. This interaction was not diffuse but confined to discrete lesion-associated niches, where it was sustained by coordinated NF-κB and TGF-β transcriptional programs. Translating these findings into a fibroblast differentiation-based risk model allowed prospective identification of severe injury states in both tissue and peripheral blood. Moreover, NT3-chitosan treatment was associated with suppression of pathological fibrosis-related transcriptional programs and improved functional outcomes, findings that are consistent with modulation of the Spp1-Itgav/Itgb1-associated niche but do not by themselves establish direct receptor blockade.

The prominence of Spp1–Itgav/Itgb1 communication between activated microglia and fibroblasts adds mechanistic depth to current models of scar formation in the central nervous system. Previous lineage tracing work showed that pericytes and perivascular fibroblasts are the cellular origins of fibrotic scarring (4, 5), yet the instructive cues that govern their activation have remained poorly defined. The four-dimensional spatial atlas shows that *Spp1* expression in microglia and *Itgav*/*Itgb1* expression in fibroblasts converge spatially at the lesion core during the peak phase of scar consolidation. This supports the idea that osteopontin-integrin signaling links immune surveillance to matrix deposition, consistent with recent evidence that microglia-derived sphingosine-1-phosphate promotes pericyte migration (6). However, our findings established *Spp1* as a more comprehensive fibrosis-instructive signal. In parallel, recent studies have highlighted the heterogeneity of fibroblasts within the fibrotic scar, revealing distinct subpopulations with specialized functions in matrix remodeling and immune crosstalk (18). This heterogeneity aligns with our observation of multiple fibroblast states in the spatial atlas. Our SCENIC analysis further identified *Nfkb1* and *Smad3* as shared regulatory hubs in both microglia and fibroblasts, suggesting that the two populations are transcriptionally synchronized rather than operating through independent programs. This finding is in line with studies showing that the NF-κB/DRP1 axis can modulate microglial polarization (19, 20) and that TGFβ1/SMADs signaling is a key driver of fibrosis after SCI (21). The coordinated enrichment of inflammatory and profibrotic regulons points to a feed-forward loop in which immune activation reinforces stromal remodeling, ultimately stabilizing a chronic lesion state that resists spontaneous repair.

We then explored whether fibroblast differentiation trajectories could be used to build a clinically relevant risk stratification tool. The fibroblast differentiation-based SCI classification effectively separated SCI samples by injury severity, treatment response and immune infiltration patterns. Cluster 3 was enriched for ASIA grade A cases and showed elevated *Itgav* expression. When condensed into a continuous fibroblast score, this signature retained discriminatory power across independent cohorts despite differences in species, platform and tissue source, indicating that the underlying biological signal is robust. A risk prediction model based on fibroblast differentiation-associated signatures in peripheral blood samples predicted ASIA grade A with good discriminatory performance, achieving AUCs of 0.883, 0.939, and 0.807 in the total, training, and test sets, respectively. This finding suggests that circulating transcriptional changes mirror central injury-associated molecular states, in line with systemic inflammatory remodeling after SCI (12). Importantly, these PBMC-based fibroblast differentiation-associated signatures should be interpreted as surrogate molecular programs in circulating blood cells. The utility of blood-based biomarkers for SCI severity has gained traction, with studies demonstrating that transcriptomic changes in peripheral blood cells can have diagnostic and prognostic value (22). Our work showed that a fibroblast differentiation-associated signature detectable in PBMCs can serve as a minimally invasive surrogate for lesion-associated fibrotic programs. Such a framework could enable early identification of patients at high risk of excessive fibrosis and guide the delivery of scar-targeted interventions before chronic consolidation takes hold.

The therapeutic efficacy of NT3-chitosan in suppressing fibrotic remodeling provides proof-of-concept support for modulation of the Spp1--Itgav/Itgb1-associated fibrotic niche. Molecular docking predicted a potential interaction of chitosan with the Itgav/Itgb1 complex, and *in vivo* treatment was associated with restored anatomical continuity, reduced scar tissue, and improved locomotor recovery in a complete transection model. Importantly, NT3-chitosan was associated with coordinated suppression of inflammatory response, TGF-β signaling, IL6-JAK-STAT3 signaling, and TNFalpha signaling via NF-kB, while enriching hedgehog signaling. This profile points to a systems-level shift rather than isolated pathway inhibition (23). It is consistent with destabilization of the chronic scar-forming milieu identified in our spatial atlas. The durability of functional gains over 52 weeks argues against transient anti-inflammatory effects alone. Prior work from our group demonstrated anti-inflammatory properties of NT3-chitosan (11), yet the present study mechanistically links those effects to integrin-mediated stromal programs. Our previous work also demonstrated that NT3-loaded chitosan can inhibit neuronal cuproptosis, adding another layer to its therapeutic mechanism (24).

Several limitations should be acknowledged. First, all animal experiments were performed in female rodents; because SCI is more common in males and sex differences influence immune and fibrotic responses, the generalizability of our findings across sexes remains to be established. Second, although our spatial transcriptomic analyses defined major lesion compartments, they did not resolve subcellular interactions, and histological markers such as IBA1 and CD68 cannot reliably distinguish resident microglia from infiltrating macrophages after SCI. Thus, while single-cell and spatial transcriptomic data support a predominantly microglia-associated signaling source, some histologically defined myeloid cells may include both populations. Validation with higher-resolution spatial approaches and microglia-specific markers such as *P2ry12* and *Tmem119* will be important. Third, although the Spp1-Itgav/Itgb1 axis was consistently prioritized across datasets and NT3-chitosan was associated with suppression of related downstream programs *in vivo*, we did not perform direct binding, receptor-blocking, recombinant SPP1 stimulation, or *in vivo* loss-of-function experiments. Our conclusions are therefore mechanistically suggestive but not yet causal. In addition, the PBMC-based fibroblast differentiation-associated signature likely reflects shared inflammatory programs rather than circulating mature fibroblasts, and the risk model requires validation in larger multicenter cohorts. Finally, translation of NT3-chitosan from a rodent transection model to human contusion SCI, particularly cervical injury, will require further study.

Future work should use single-nucleus ATAC-seq or CUT&Tag profiling to resolve the chromatin landscape of scar-forming fibroblasts. Longitudinal imaging of the fibrotic niche could define how microglia–fibroblast communication evolves from acute coupling to chronic scar maintenance (25). Clinically, integrating the fibroblast risk score into neurological assessments may improve early prognostication for anti-fibrotic therapies.

## 5 Conclusions

Our findings establish microglia-fibroblast crosstalk via the Spp1-Itgav/Itgb1 axis as a central driver of fibrotic scar maturation after SCI. The integrated 4D spatial atlas and fibroblast differentiation-based SCI classification provide a clinically actionable framework for risk stratification and targeted intervention.

## Data Availability

The datasets presented in this study can be found in online repositories. The names of the repository/repositories and accession number(s) can be found in the article/**Supplementary Material**.

## Supplementary material

Supplementary Figure 1 Temporal dynamics of immune-stromal ligand-receptor networks after SCI. **(A)** Circle plot depicting the number of inferred interactions between cell groups at 7 dpi. **(B)** Interaction strength heatmap identifying activated microglia and fibroblasts as major hubs. **(C)** Ligand-receptor bubble plot highlighting interactions from activated microglia and fibroblasts to other cells at 7 dpi. **(D)** Ligand-receptor bubble plot highlighting interactions from other cells to activated microglia and fibroblasts at 7 dpi. **(E)** Circle plot depicting the number of inferred interactions between cell groups at 2 months post-SCI. **(F)** Interaction strength heatmap at 2 months post SCI. **(G)** Ligand-receptor bubble plot highlighting interactions from activated microglia and fibroblasts to other cells at 2 months. **(H)** Ligand-receptor bubble plot highlighting interactions from other cells to activated microglia and fibroblasts at 2 months.

Supplementary Figure 2 Independent single-cell validation of the microglia-fibroblast communication axis. **(A)** UMAP integration of GSE162610 cells across conditions. **(B)** UMAP of 23 cell clusters. **(C)** Dot plot of cluster-enriched genes. **(D)** Consolidation into 15 major cell types. **(E)** Heatmap of marker gene expression among 15 major cell types. **(F)** Cellular composition changes after SCI. **(G)** Stacked bar plot showing the distribution of injury stages across different cell types, with upper scatter plot displaying the expression of core profibrotic signature genes. **(H)** Cell-cell interaction network among 15 major cell types. **(I)** Ligand-receptor network among 15 major cell types. **(J)** Reclustering of fibroblasts identifies seven subclusters. **(K)** Annotation of fibroblast subclusters. **(L)** Differential expression among fibroblast subclusters. **(M)** Marker heatmap of fibroblast subclusters. **(N)** Abundance changes of fibroblast subclusters over time.

Supplementary Figure 3 Transcriptional and cell-cycle heterogeneity underlying fibroblast differentiation states. **(A)** Cell-cycle projection of fibroblasts. **(B)** G2M and S phase scores across clusters. **(C)** iTALK ligand-receptor network among fibroblast subclusters. **(D)** Interaction landscape dominated by ECM and adhesion signaling. **(E)** Monocle2 branched fibroblast trajectory. **(F)** Pseudotime mapping of fibroblast subclusters. **(G)** State annotation along trajectory. **(H)** Pathway enrichment of state-associated genes. **(I)** Branch-dependent gene modules of fibroblast differentiation trajectory. **(J)** Correlation matrix of trajectory gene modules. **(K)** Consensus clustering in GSE69334 identifies three subtypes. **(L)** Consensus matrix showing cluster stability in GSE69334. **(M)** Consensus clustering in GSE151371 reproduces three subtypes. **(N)** Consensus matrix showing cluster stability in GSE151371.

Supplementary Figure 4 Clinical and biological stratification of SCI by fibroblast differentiation-based subtypes. **(A)** Association of three subtypes with post-injury time, treatment, injury status and ASIA grade. **(B)** Subtype-specific expression patterns, including Itgav. **(C)** Spatiotemporal stratification of the transcriptional program. **(D)** Fibroblast scores increase progressively from Cluster 1 to Cluster 3. **(E)** Expression of *Col1a2*, *Dcn*, *Jun* and *Cd68* in fibroblast-high versus fibroblast-low groups. **(F)** PCA of three subtypes in GSE69334. **(G)** PCA of three subtypes in GSE151371. **(H)** Fibroblast-score enrichment at early and intermediate time points in GSE69334. **(I)** Sankey analysis of subtype, fibroblast-score state, treatment and time in GSE69334. **(J)** Expression levels of differentiation-associated genes across different subtypes in GSE69334. **(K)** Immune infiltration differences among different subtypes in GSE69334.

Supplementary Figure 5 Peripheral blood fibroblast differentiation-associated signatures predict severe neurological impairment. **(A)** Differential expression of fibroblast differentiation-associated genes across time, treatment and anatomical location. For each region (rostral, middle, caudal), n = 3 pooled samples per treatment group at each time point. Total samples: rostral n = 63, middle n = 38, caudal n = 63. Mean ± SEM. One-way ANOVA with Bonferroni *post hoc* test. **(B)** Volcano plot of differentially expressed genes between SCI and uninjured samples. Uninjured: n = 3; SCI: n = 164. Limma with Benjamini-Hochberg FDR correction. |log_2_FC| > 0.5, FDR < 0.05. **(C)** Differentially expressed transcription factors across rostral (n = 63), middle (n = 38), and caudal (n = 63) regions. Mean ± SEM. One-way ANOVA with Bonferroni *post hoc* test. **(D)** GO enrichment of fibroblast differentiation-associated genes. **(E)** Expression of fibroblast differentiation-associated genes in peripheral blood from ASIA grade A (n = 23) vs. non-A (n = 37) patients (GSE151371). Mean ± SEM. Unpaired two-tailed Student’s t-test. **(F)** LASSO regression for risk prediction model. Training set: n = 100; test set: n = 64. **(G)** Signature gene expression between high- and low-risk groups. Total set: n = 164; training set: n = 100; test set: n = 64. Mean ± SEM. Unpaired two-tailed Student’s t-test. **P* < 0.05, ***P* < 0.01, ****P* < 0.001. **(H)** ROC curves for predicting ASIA grade A. Total set: n = 164, AUC = 0.883; training set: n = 100, AUC = 0.939; test set: n = 64, AUC = 0.807. **(I)** Immune infiltration scores in high-risk (n = 82) vs. low-risk (n = 82) samples. Mean ± SEM. Unpaired two-tailed Student’s t-test. **P* < 0.05, ***P* < 0.01, ****P* < 0.001. **(J)** GSVA hallmark pathway enrichment in high-risk (n = 82) vs. low-risk (n = 82) samples. Limma with Benjamini-Hochberg FDR correction.
